# Same Game, Different Players: Emerging Pathogens of the CF Lung

**DOI:** 10.1128/mBio.01217-20

**Published:** 2021-01-12

**Authors:** Alexa D. Gannon, Sophie E. Darch

**Affiliations:** a Department of Molecular Medicine, Morsani College of Medicine, University of South Florida, Tampa, Florida, USA; b Department of Internal Medicine, Morsani College of Medicine, University of South Florida, Tampa, Florida, USA; University of Texas Health Science Center at Houston

**Keywords:** cystic fibrosis, emerging pathogens, microbial interactions

## Abstract

The survival rates of individuals with cystic fibrosis (CF) have significantly increased as a result of improved therapies, such as the inclusion of cystic fibrosis transmembrane conductance regulator (CFTR) modulators for some mutations. However, microbial infection of the airways remains a significant clinical problem. The well-known pathogens Pseudomonas aeruginosa and Staphylococcus aureus continue to establish difficult-to-treat infections in the CF lung. However, in recent years, there has been an increased prevalence of both Aspergillus fumigatus (*Af*) and non-tuberculous mycobacteria (NTM) species isolated from CF patient sputa. The emergence of these pathogens opens an important area of discussion about multikingdom infections, specifically, how interspecies interactions have the potential to shape the course of infection, such as tolerance to host immune defenses and antimicrobial therapies. Their ability to establish themselves in an existing polymicrobial environment suggests to us that microbial interactions play a significant role, and characterizing these mechanisms and understanding their implications will be critical to the future development of better antimicrobial therapies. With this minireview, we hope to inspire conversations about and demonstrate the merit of more research in this area.

## Abstract (precis)

Incidences of non-tuberculosis mycobacteria (NTM) and Aspergillus fumigatus have increased around the world over the past decade and have become a significant health threat to immunocompromised individuals such as those with cystic fibrosis (CF). CF is characterized by the buildup of mucus in the lungs which become chronically infected by a myriad of pathogens.

## INTRODUCTION

The survival rate of individuals with cystic fibrosis (CF) has increased considerably as a result of improvements in clinical care, as well as the introduction of successful modulator therapies for a subset of cystic fibrosis transmembrane regulator (CFTR) mutations. However, microbial infection of the airways remains a significant clinical problem and leading cause of mortality (1). The CF lung houses a complex community of microbes, highlighting a successive relationship between age and cultured microbes from sputum. Pseudomonas aeruginosa (*Pa*) continues to be the predominant pathogen in the adult CF lung, where it establishes antimicrobial-resistant, difficult-to-treat infections (2, 3). The presence of Burkholderia cepacia complex (*Bcc*) in the CF lung has long been acknowledged as the greatest threat to lung health in early adulthood; however, with increased life expectancy, new infectious agents are beginning to emerge. In recent years, there has been an increased global prevalence of both non-tuberculous mycobacteria (NTM) and Aspergillus fumigatus (*Af*), a pathogenic fungus, isolated from CF patient sputa (1, 2, 4). Although much is known about these organisms individually, their role in the CF lung is relatively unknown—including the consequences of their increasing presence in the clinic.

The emergence of these pathogens opens an important area of discussion about multikingdom infections. Specifically, understanding the potential that interspecies interactions have to shape the course of infection in CF—such as tolerance to antimicrobial therapies and host immune defenses. It also highlights the limitations of current models for observing these interactions. Traditional approaches to studying chronic infections are often based on (i) single species communities and (ii) the belief that the behavior of an individual cell is representative of its behavior as a group, despite growing evidence that shows this is not the case.

We now know that bacteria have mechanisms for social interactions that allow them to live in structured, multispecies communities (5). These communities are like any other ecosystem, where a complex network of relationships exists between the various inhabitants, ranging from cooperative to competitive. There are also many well-documented examples of interspecies and interkingdom synergistic microbial behaviors, and the complex microbiome of the CF lung provides an environment which can be modeled in order to study such interactions in the context of human disease (6, 7). Although many aspects of these emerging pathogens remain to be understood on a single species level, their ability to establish themselves in an existing polymicrobial environment in the CF lung strongly suggests that microbial interactions play a significant role in both disease progression and pathogenesis.

## INTRODUCING THE EMERGING PATHOGENS

### (i) Non-tuberculous mycobacteria (NTM).

NTM are ubiquitous atypical Gram-positive bacteria found in soil and water that can cause chronic, difficult-to-treat pulmonary disease (other than tuberculosis) in humans. NTM have been shown to form biofilms *in vivo*, most commonly associated with respiratory infections (8, 9). While NTM primarily cause infection in immunocompromised populations, most notably in CF, they can also cause infections in otherwise healthy individuals. Global rates of NTM infection in both healthy and immunocompromised populations have risen in the past decade, but it is unclear whether this increase is the result of improved surveillance or an actual increase in prevalence (10, 11). Many agree that NTM pose a significant health threat to those with CF, but the community lacks a consensus about risk factors, clinical outcomes, indications for lung transplant, and best practices for screening and treatment. The role NTM play in the CF lung is also not well understood; having a positive culture (especially at low CFU) is not sufficient for diagnosis of NTM pulmonary disease (NTM-PD) (12). In fact, studies have shown that a number of patients who culture positive for NTM will have a negative culture soon after, suggesting the possibility of transient colonization (13). The ability of NTM to establish colonization but then be removed from the airways, especially in immunocompromised individuals like those with CF, suggests that more than just the host immune system is involved. A possible explanation is that predatory behaviors of other resident microbes may play a role in population dynamics where some organisms transiently occur over time.

Within the CF population, two species of NTM predominate in 95% of NTM infections. These are Mycobacterium abscessus complex (MABSC) which includes the species M. abscessus, M. massiliense, and M. bolletii, and Mycobacterium avium complex (MAC), which is comprised of M. intracellulare, M. hominissius, and four M. avium subspecies (14, 15). Species prevalence varies between geographical regions; MABSC and other non-MAC NTM are most commonly isolated from patients in Europe and Israel, while MAC is most common in North America. The rapid growing MABSC is considered more virulent and harder to treat than its slow growing relative MAC; when grown on NTM-selective media, MABSC requires a 10-day incubation, while MAC cultures must be incubated for 28 days (16). As many as 50 to 80% of MABSC culture-positive CF individuals develop invasive NTM lung disease, while this is seen in less than half of MAC culture-positive patients (17). This increased virulence is thought to contribute to MABSC acquisition early in life; multicenter studies have shown MABSC-positive cultures from young children with prevalence peaking between the ages of 11 and 20 years, whereas MAC is rarely seen in children under 5 and peaks in prevalence after age 25 (14, 18, 19). Chronic MABSC infection has been repeatedly linked with decreasing lung function, failed culture conversion, and higher risk of developing NTM pulmonary disease (NTM-PD). In comparison, MAC-positive cultures have not been consistently correlated with a decline in lung function (19).

NTM have many unique characteristics that make it difficult to treat in any patient population. In immunocompromised individuals with CF, these are compounded with chronic inflammation, lung remodeling, and coinfections with other organisms (13, 20). Mycobacteria derive their name from the mycolic acids found in their cell wall which form a hydrophobic layer over peptidoglycan and the plasma membrane. These long-chain fatty acids give the cell wall more rigidity and protection from antimicrobials than is seen in most Gram-positive species, where the peptidoglycan layer is exposed (21). The unique composition of the mycobacterial cell wall offers intrinsic protection in many hostile environments—such as those created by the host immune response and many antimicrobials—which makes NTM infection notoriously difficult to treat. MAC and MABSC have also been shown to form biofilms both *in vitro* and in the CF lung, which have a demonstrated role in their resistance to treatment and modulation of the host immune response (8, 9, 15). Additionally, coinfections with NTM and other organisms associated with CF, such as Pseudomonas aeruginosa and Aspergillus fumigatus, have been consistently linked to poorer clinical outcomes (13, 20).

Treatment for NTM infection is long and complex. Both MAC and MABSC require multidrug regimens that include a macrolide (usually azithromycin) and often last for many years (19). MAC infections are usually treated with a course of three antibiotics, one being a macrolide. MABSC infections are more difficult to treat; treatment is applied in two phases and involves a combination of intravenous (IV), oral, and nebulized antibiotics (17). However, the global CF community has seen increasing incidences of macrolide resistance among MABSC species that have a functional erythromycin ribosomal methylase (*erm*) gene or point mutations in the 23S rRNA. Macrolides like azithromycin are common components of CF treatment regimens and NTM-specific treatments, so this acquired resistance has made antimicrobial monotherapies untenable for CF patients (19).

There is a great deal of controversy among clinicians on the subject of NTM infection as a contraindication for lung transplant. Specifically, many centers consider Mycobacterium abscessus a contraindication, as it is known to cause post-surgical wound infections, but will allow transplants in patients with MAC (22). MABSC culture conversion in CF patients is rare; however, the reservoir(s) for MABSC in postsurgical infections have not been precisely identified (17). In single lung transplants, there is always the possibility that the remaining lung harbors numbers of NTM that are either too low to detect or avoid detection because they are embedded in the tissue (9). Similarly, it is worth considering soft tissues and other organs as possible reservoirs, as MABSC can cause infection in a multiple sites (23). Finally, it is also possible that patients who develop post-transplant MABSC infections acquired it from the environment, often due to a higher susceptibility of individuals as a result of immune-suppressive drugs given to prevent transplant rejection. Despite MABSC’s propensity for causing postsurgical infections, the presence of NTM does not uniformly correlate with the development of NTM pulmonary disease. A 15-year cohort study showed that while it was common for lung transplant recipients who were previously NTM culture negative to become culture positive (22.4%), NTM did not progress to NTM-PD in a majority of patients (24). The presence of NTM in post-transplant patients without disease shows that NTM-PD can be successfully managed with early and aggressive treatment.

### (ii) Aspergillus fumigatus (*Af*).

A. fumigatus is a filamentous fungus that has recently been recognized as an emerging health threat to those with CF. It has become one of the most common pathogens in the pediatric CF population, suggesting that *Af* colonization occurs at a young age (3, 25). *Af* does not normally cause disease in healthy populations, but it can become invasive in immunocompromised patients like those with CF. Its effect is twofold: *Af* can cause chronic infections after colonizing the lung, and it is also responsible for an allergic disease termed allergic bronchopulmonary aspergillosis (ABPA). ABPA is a hypersensitive allergic reaction that causes the activation of TH2 helper T cells, recruitment of eosinophils, and increased inflammation (26). ABPA can progress to invasive or chronic pulmonary aspergillosis, and in extreme cases, it can lead to the formation of aspergillomas or central nervous system aspergillosis (27, 28).

CF sputa may be culture positive for *Af* without any clinical presentation of ABPA in that individual, but there are differing opinions about the independent effect of non-ABPA *Af* infections on lung function and pulmonary exacerbation. A large retrospective study found CF patients with chronic *Af* infections scored 3.61% lower on forced expiratory volume (FEV) tests than those who were uninfected and that coinfections of *Af* and *Pa* had a significant negative effect on lung function. The authors concluded that chronic *Af* infection other than ABPA is an independent risk factor for hospitalization for individuals with CF (29). Interestingly, a different study the following year did not find an association between *Af* infection and decreased lung function in CF where a culture-positive sputum for *Af* was detected without having ABPA (30). More recently, the presence of *Af* in sputum of patients without ABPA was associated with worse respiratory function and lower quality of life. These studies underscore the disparities in our knowledge of the impact of *Af* across the CF population and solidify the need for better understanding of *Af* in the environment of the CF lung (31).

Unlike most other pathogens traditionally associated with CF, *Af* is a eukaryotic organism. This presents its own treatment challenges, as drug toxicity and unwanted interactions with other CF drugs are a major concern. At present, there are no clear guidelines for prevention of *Af* acquisition or treatment of *Af* that has not progressed to ABPA. ABPA can be difficult to detect in CF patients, as many of its symptoms overlap with those regularly observed in CF respiratory disease (32). Patients who have been diagnosed with ABPA are often treated with a combination of a corticosteroid and antifungal, most commonly triazoles (32, 33). An increasing prevalence of azole-resistant *Af* strains have been isolated over the past decade, which has been linked to long-term azole therapy in infected individuals and azole-based fungicides used in agriculture (34, 35). In cases of azole resistance, many experts recommend switching to treatment with liposomal amphotericin B (L-Amb) (27). However, the high costs of L-Amb therapeutics and additional need to monitor for toxicity and adverse interactions with other drugs highlights the need for the development of better therapies.

The lack of universal standards for *Af* screening and culturing poses a significant barrier to consistent and accurate diagnoses. These inconsistencies have drawn attention to the use of culture-based methods in diagnostic laboratories, which are often less accurate than molecular methods (25). A recent study demonstrated that quantitative PCR (qPCR) was up to 94.2% more likely to detect *Af* in the sputum samples of pediatric patients than traditional culture-based methods, indicating that this practice has led to an underestimation of *Af* prevalence and average age of onset of *Af* disease in CF (36). Furthering our understanding of *Af* could lead to the development of new treatment practices for young patients that are aimed at preventing *Af* colonization—similar to current practices for P. aeruginosa (*Pa*).

The presence of *Af* or even a diagnosis of ABPA is not commonly regarded as a contraindication for lung transplant in CF; however, a lung transplant is not always considered a curative treatment for *Af*. Indeed, *Af* is the most common cause of post-transplant fungal lung infections in both CF and non-CF individuals, regardless of detectable colonization before transplant (37). A study of CF individuals colonized with *Af* pre-transplant showed that nearly 60% were culture positive post-transplant and that treatment with prophylactic antifungals did not prevent the development of tracheobronchial aspergillosis (TBA) (38). There is a significant relationship between post-transplant *Af* infection and both acute and chronic rejection, as well as an association between *Af* infection and 5-year post-transplant mortality rates, emphasizing the need for improved detection and therapeutics of *Af* in the context of CF (39).

## POTENTIAL FOR INTERACTIONS IN A POLYMICROBIAL COMMUNITY

Very little is known about interactions between these organisms relative to the CF lung, a fact that prompted us to write this minireview. We hope to demonstrate the merit of this line of research and encourage future exploration of the subject.

### (i) NTM and Aspergillus fumigatus *(Af)*.

At present, there are few studies that focus on interactions between NTM and other organisms in any infection model. To our knowledge, there are no studies to date that explore a relationship between *Af* and NTM. However, both NTM and *Af* have independently been associated with poor health outcomes in CF patients (19, 31). Clinical recommendations for the management of NTM in CF patients consistently link NTM-positive cultures and NTM-PD with the presence of *Af*, and CF patients with ABPA have been shown to be >2 times more likely of producing an NTM-positive culture (19, 40). These findings strongly suggest an understudied relationship between NTM and *Af* that has clinical implications for CF patients.

### (ii) Pseudomonas aeruginosa (*Pa*) and Aspergillus fumigatus (*Af*).

*Pa* is one of the most pervasive pathogens in the CF airway and is a leading cause of mortality. As such, many of the interspecies interactions that have been characterized in the CF airway involve *Pa*. *Af* is one of the most common fungal pathogens recovered from the CF lung, where cocolonization of *Pa* and *Af* has been strongly correlated with a worse prognosis (41). Because of this, there has been increasing interest in its relationship with *Pa*. The full nature of the interactions between *Pa* and *Af* in the CF lung is yet to be completely understood, but current data suggest that it is more complex than was previously believed.

**FIG 1:**
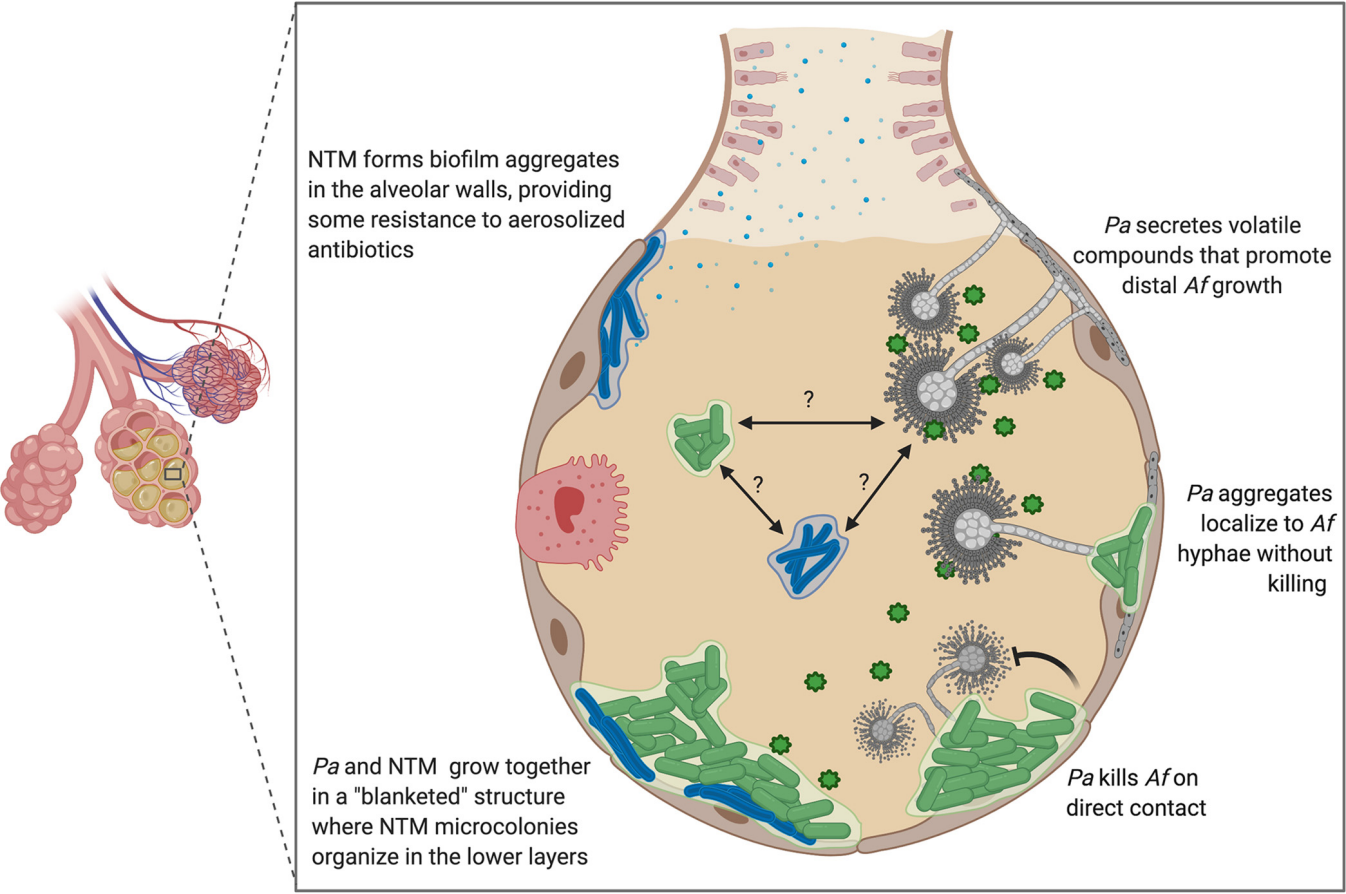
Proposed ecology and interactions of emerging pathogens in the CF lung. Possible interactions between NTM, *Pa*, and *Af* in the CF lung based on the findings of various *in vitro* studies. *Pa* and NTM have been shown to form multispecies biofilms *in vitro* increasing antibiotic resistance in both organisms (6). *Pa* and *Af* have shown both synergistic and predator-prey interactions *in vitro*, which could serve to maintain community boundaries *in vivo* (7, 43, 65). *Pa* found in the CF lung is frequently observed as aggregates of cells, which often exhibit behaviors different to petri dish or flow cell biofilms (44). Preliminary data (Darch lab) indicate that *Pa* aggregates may localize to *Af* hyphae without a killing effect. This figure was created with BioRender.com.

*Af* and *Pa* inhabit similar niches in both nature and human infection, so the idea that both organisms have developed adaptations to compete against one another for shared resources is not surprising. Indeed, there is a well-documented antagonistic relationship between *Af* and *Pa*, in which *Pa* has been shown to inhibit *Af* biofilm formation and kill *Af* cells on contact (42, 43). However, like many other organisms with a shared ecology, they are capable of more than just predator-prey interactions. Recent studies have revealed a surprising synergistic relationship between *Pa* and *Af*, in which *Pa* was shown to support the growth of *Af* outside its immediate area by emitting volatile gaseous compounds that *Af* can utilize as metabolites (Fig. 1) (7). Taken together, these findings clearly demonstrate the importance of spatial organization within polymicrobial communities and show that microbial social interactions do not occur in just one dimension.

All of the studies we reference here found predatory or synergistic interactions between *Pa* and *Af* that were grown in a variety of closed laboratory systems where both organisms are able to form surface-attached biofilms (7, 42, 43). In a defined synthetic media (SCFM2) that closely mimics the environment of the CF lung, *Pa* has been shown to grow in free-floating, dense clusters of ∼10 to 1,000 cells, termed aggregates (44). Combining *Pa* aggregate growth with the characteristic hyphal growth of *Af*, the localization of *Pa* aggregates with *Af* hyphae can be observed without residual killing effects (Fig. 1; S. E. Darch, unpublished data). These preliminary data suggest that coexistence can be observed in an environment similar to the CF lung and offer the potential to understand relationships that exist between two pathogens *in vivo*. The ability to coexist raises many questions about how this relationship might impact treatment efficacy, lung function, and disease progression in CF. These data not only highlight the need for more research in this area but also demonstrate the utility of experimental systems that closely mimic host infection sites.

### (iii) Pseudomonas aeruginosa (*Pa*) and NTM.

While compiling this minireview, we found very few published studies that detail the interactions between *Pa* and NTM, although many experts agree that coinfections with these organisms are associated with negative patient outcomes both in CF and non-CF populations (20, 45). Rodriguez-Sevilla et al. (6) explored the mechanisms of interactions between *Pa* and NTM, demonstrating that clinical isolates of *Pa* and NTM (M. abscessus) are able to coexist in a dual species biofilm *in vitro*, where they form a “layered” or “blanketed” structure in which the *Pa* biofilm grows on top of the NTM biofilm (Fig. 1). *Pa* contributed at a higher ratio in the biofilms; however, the ratio of both organisms was maintained steadily over the course of 72 h. The study also identified a significant decrease in NTM growth in dual species biofilm with *Pa* compared to NTM single species biofilm, which could be explained by the inherent traits of each organism. *Pa* is fast growing and motile, while NTM is relatively slow growing and non-motile. This could give *Pa* an advantage that allows it to outcompete NTM; however, these results suggest that *Pa* does not or cannot directly inhibit NTM (6). When considering whether this interaction occurs *in vivo* in the CF lungs, it would not only have important implications for treatment efficacy but also pathogen surveillance. The dual species biofilms in which *Pa* grew a “blanketed” biofilm over NTM demonstrated increased antibiotic resistance in this study—it is plausible that this structure (Fig. 1) could also protect NTM from pathogen surveillance techniques such as bronchoalveolar lavage (BAL) and contribute to its underdetection (6).

The ability of multiple organisms to establish and maintain a biofilm mode of growth has important implications for treatment, if it indeed occurs in the CF lung and is not an artifact of the lab. NTM infections in CF patients are often treated with azithromycin or clarithromycin, as they are some of the only oral antibiotics that have shown to be consistently effective against NTM species. In the study by Rodriguez-Sevilla et al. *Pa* and NTM single species biofilm were significantly reduced by clarithromycin treatment; however, dual species biofilms were unaffected (6). These data demonstrate the need for more studies to define the mechanisms of therapeutic resistance in dual species biofilms, as well as the clinical implications of when they fail.

## HOW DO WE STUDY EMERGING PATHOGENS IN EXISTING MICROBIAL COMMUNITIES?

To begin understanding the role emerging pathogens may play in CF infections and their impact on disease outcomes, we need a more complete picture of the dynamics of the polymicrobial communities they are a part of. To this end, there are several areas we as researchers need to consider. For example, how do the dynamics of the existing polymicrobial community change to allow emerging pathogens to grow? This question focuses on the potential interactions between species, that in short determine the “rules” of a microbial community. Many of the pathogens associated with the CF lung occur in loose succession over time; therefore, how emerging pathogens become established is important. Another topic to consider is the layout or geography of microbial communities within the infection site. Many interactions in polymicrobial communities are mediated by the physical space between and within individual species (Fig. 1). Examining the spatial organization of emerging pathogens within the CF lung raises questions about how the distribution of species contributes to evasion of either host or chemical interventions and the ability of existing therapeutics to target them effectively (46). Last, we should consider the interplay between these microbes and the host. How these organisms persist despite host immune defenses can contribute vital knowledge to improving treatments.

### (i) What do we know about community dynamics so far?

Like any ecosystem, the microbes frequently observed in the CF lung are linked by a complex web of interactions that are critical for community fitness. When dysbiosis occurs in a polymicrobial community, ecological niches open that allow new organisms to grow and existing populations to expand beyond their previous numbers. One of the most frequently described occurrences of such “displacement” is that which can be observed between Staphylococcus aureus (*Sa*) and *Pa* in the CF lung. The interactions between these two organisms have been extensively studied by many laboratories, providing a platform for many of the future research avenues we discuss in this minireview. Previous studies have demonstrated an abundance of *Sa* infections in children, followed by an established *Pa* infection by early adulthood (47, 48). However, in recent years, this relationship has been the subject of increasing discussion. According to the *Cystic Fibrosis 2018 Patient Registry Annual Report*, the number of patients with *Pa* infections has shown a continual decline over time, with the sharpest decrease in *Pa* infection prevalence seen in patients under 20 (1). At the same time, the presence of *Sa* in the CF lung is often observed well into early adulthood at some baseline level. These changes are thought to be due in large to improved therapeutic regimens and early eradication practices implemented in children with CF. However, this progress has been paralleled by a global rise in prevalence of both *Af* and NTM infections (3, 4). There are many unknown factors driving the emergence of these pathogens, highlighting the need to understand how the presence or absence of other microbes in the CF lung may influence susceptibility to emerging pathogens.

### (ii) Community changes as a result of improved therapeutic interventions.

CF clinics around the world have observed shifts in the epidemiology of CF lung infections over the past decade (49). Current treatment guidelines for young children with CF focus on delaying or preventing the establishment of chronic *Pa* infections, which has led to a lower prevalence of *Pa* infection among young CF patients (3). A study that followed CF patients as they transitioned from pediatric to adult care found lower rates of *Pa* infection but increasing rates of both *Af* and NTM infections in patients who had undergone eradication treatment for *Pa* infections as children (2). As a fast growing, motile organism that has been shown to outcompete *Af* and NTM in certain conditions, it is possible that aggressive *Pa* eradication treatment could inadvertently create a niche for emerging pathogens like NTM and *Af* (6, 42). Although there are suggestions that increasing prevalence of these pathogens can be attributed to improved surveillance and screening methods, this dynamic switch correlated with therapeutic interventions highlights an important observation that needs to be further understood (36, 50).

Within the studies that have reported shifts in the well described “microbiome” of the CF lung over the past 2 decades, only some include the recent increasing occurrence of both NTM and *Af* (1, 3). However, there is a significant gap in knowledge of both the abundance and dynamic shift (if any) of either organism. Given this, for NTM and *Af* infection in the context of CF, it is difficult to know whether they displace other known CF pathogens when establishing infection or fill vacant niches potentially created by therapeutic treatment. An alternative theory is that these pathogens have existed commensally in the CF lung and have remained undetectable (36, 50). Surveillance efforts for the detection of both NTM and *Af* have increased, although they are not always applied uniformly. This is primarily due to current clinical recommendations that often advise against the use of less invasive oropharyngeal swabbing for sample collection to detect the presence of NTM and *Af* (19, 25). Culture-based screening methods for both organisms continue to dominate, despite a growing body of work demonstrating their lack of sensitivity for *Af* in sputum (36). Moving forward, practices will likely include a mixture of culture-based and molecular approaches, for which there is plausible support for both.

### (iii) How are microbial communities assembled in the CF lung?

There has been a shift in recent years away from the “classical” perception of microbial communities as single species colonies that exist in isolation from other organisms. We now know that most microbes live in mixed polymicrobial communities and that these communities are not randomly assembled—their structures suggest ordered organization (51). The spatial organization of and within microbial communities is a modern example of the old adage “form follows function.” Many studies have shown that the positioning and proximity of organisms influence inter- and intraspecies interactions and microbial behaviors, such as quorum sensing, secretion of inhibitory molecules, regulation of virulence factors, and biofilm formation (Fig. 1) (6, 43, 52–54). This in turn impacts disease progression and treatment outcomes. In the face of increasing antibiotic resistance and emerging pathogens, the development of novel therapies will benefit greatly from the use of infection models that consider spatial organization.

Microbial spatial organization in CF airways can be examined from a macroscale (whole lung) to the microscale. From the macroscale perspective, the physical location of a pathogen in the lung dictates the other microbes that are available to interact with and is well defined for many pathogens in the CF airway. There is evidence that microbial taxa within the CF lung inhabit distinct regions in different lobes of the lung with limited interactions between lobes, which has been correlated as a driver of strain diversification (46). The upper airways indicate greater population by aerobic pathogens, while the lower airways are home to fungi and many facultative and strict anaerobes, including *Af* and NTM (9, 55). These findings suggest a relationship between microbe location in the lung and treatment outcomes. For example, aerosolized antibiotics are often less effective at treating facultative and obligate anaerobes and biofilm formers in the lower lung where oxygen concentrations are lower. Location within the lung is also important to consider when characterizing clinical samples, as many common sampling techniques, such as expectorated sputum, may not give an accurate representation of microbes from the lower airways.

Spatial organization at the microscale also has a demonstrated effect on microbial fitness, but the exact mechanisms of this effect are just beginning to be explored. The concentrations of compounds such as oxygen, nutrients, signal molecules, and secreted antimicrobials can vary dramatically over just micrometers in distance. These gradients may be created by host factors or other microbes and have been shown to influence bacterial organization, gene expression, and behaviors (52, 56–58). For example, many studies have found evidence that various interactions between anaerobic and aerobic bacteria in the CF lung enhance the fitness and pathogenicity of the latter (54). Anaerobes produce by-products such as short-chain fatty acids, amino acids, and beta-lactamases that can be utilized by other pathogens; thus, pathogens that are spatially oriented to reap this benefit may have a fitness advantage over others that are not. Other data indicate that anaerobic production of a specific fatty acid, 2,3-butanediol, promotes *Pa* biofilm formation and enhances virulence, an example of how spatial orientation can modulate bacterial behavior (59–61).

There are many strengths and weaknesses in the resources available to us as researchers when we consider the most relevant model or clinical sample to study spatially organized microbial communities. At the microscale, the mode of growth adopted by an organism should be considered. Recent studies have acknowledged the existence of aggregates, small clusters of cells of ∼10 to 1,000 cells, which have been observed in both CF lung tissue and CF sputum (62, 63). How and why these structures form is not known, although host proteins, extracellular DNA, and the physical parameters of the infection site likely shape their formation. We have learned a lot from the study of explanted tissues in CF, providing important benchmark data about end stage disease. To learn more about the path that leads to these snapshot findings, we must use a combination of models and approaches to address the interactions that contribute to the data we have thus far.

### (iv) Host-microbe interactions.

While the importance of pathogen-pathogen interactions in disease states is gaining appreciation, another key factor that should be considered is how these organisms and the host interact. Host interactions have been shown in multiple systems to influence strain diversification and disease progression, indicating that understanding these mechanisms will be critical to the development of new therapies. In the context of CF, the hallmark immunological responses such as inflammation and lung remodeling are well documented and have the potential to exert selective pressure on resident bacteria. As in other ecosystems, the environment can be a driver of phenotypic diversity. *Pa* isolated from CF lungs shows a high degree of diversity between strains of the same ancestor, including genes for antibiotic resistance; it would be expected to find this in other CF pathogens as well (5, 46). In contrast, pathogens also have direct effects on the host. For example, anaerobic bacteria may promote neutrophil recruitment by releasing short-chain fatty acids that stimulate lung epithelial cells to release proinflammatory cytokines (54). This in turn likely has effects on other resident pathogens, quickly revealing a complex ecosystem network of relationships.

Characterizing interactions between the host and a single pathogen, for example between *Pa* and host neutrophils, provides important baseline knowledge, but the polymicrobial nature of most infections means that this approach does not reveal a complete picture. Approaching pathogen-host interactions from the perspective of a shared ecology requires robust models that recreate *in vivo* conditions or can be used *in situ.* Methodologies like transcriptome sequencing (RNA-seq), proteomics, and metabolomics are powerful tools to this end because they allow us to profile both host and pathogen in a variety of conditions. A recent study by Margalit et al. used proteomics to reveal a surprising relationship between *Af*, *Pa*, and host epithelial cells that contributes to *Pa* proliferation (64). They found that epithelial cells exposed to *Af* or *Pa* exhibited unique proteomes, which were also distinctly different from the proteome of cells exposed to both pathogens sequentially. Moreover, they found that exposure to *Af* made cells unable to phagocytize bacteria, thereby creating an environment more favorable to *Pa* (64). While these techniques have great utility in discerning host-pathogen interactions, they are still limited by the *in vitro* methods used to cultivate the cells. The CF community lacks *in vitro* models that robustly recapitulate the environment—and therefore gene expression—in the CF airways.

## STUDYING POLYMICROBIAL COMMUNITIES IN A 3D SPACE

**FIG 2:**
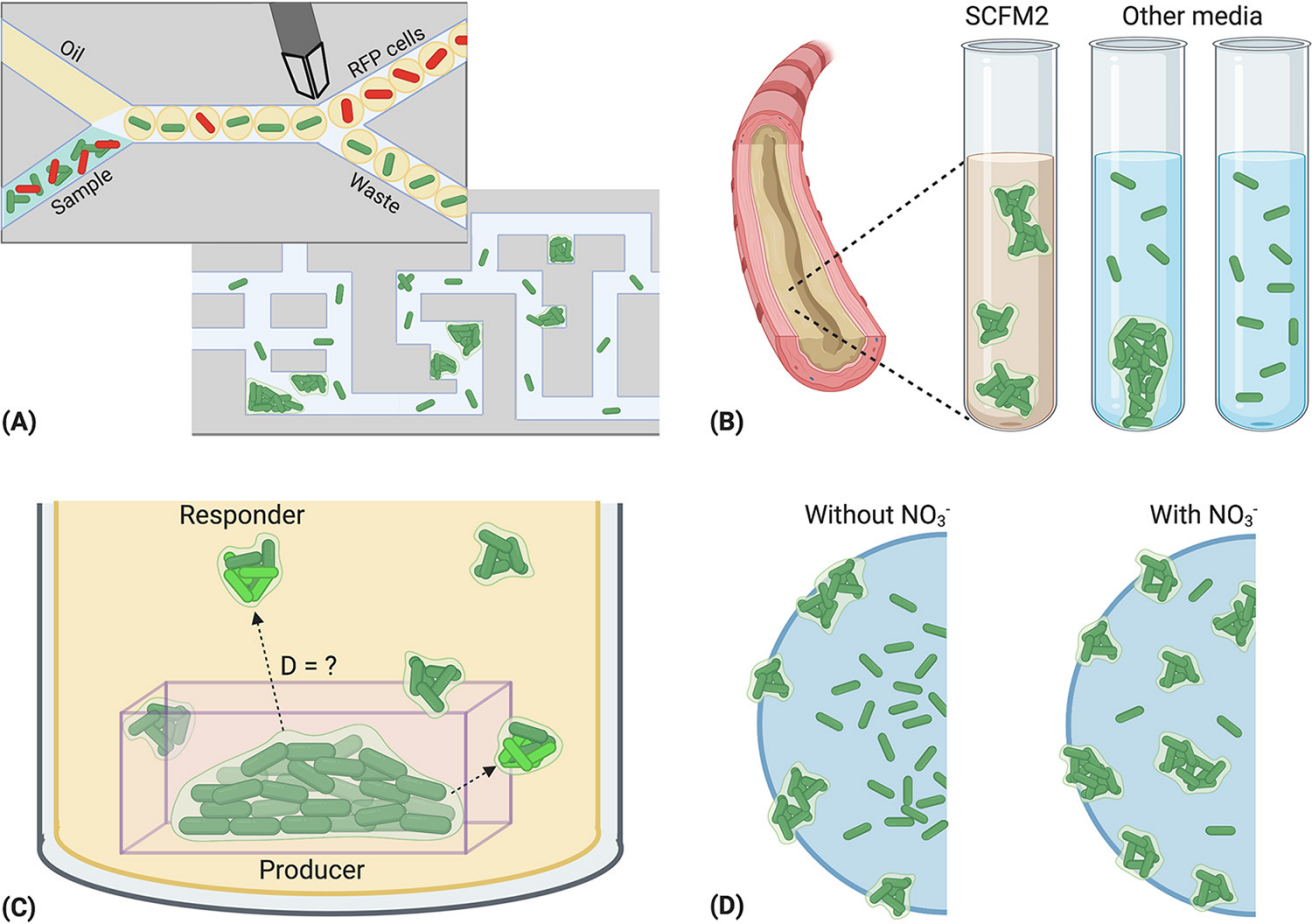
Examples of experimental methods used to study bacterial interactions at high resolution. (A) Droplet microfluidics provide a high-throughput platform for studying populations of cells. Droplets are often formed by flowing oil and bacteria-containing media through a T-shaped junction within a chip, resulting in small media droplets suspended in oil. Droplet size and bacterial load are customizable, allowing for the isolation of single cells. Cells are often labeled with a fluorescent reporter to allow visualization or sorting. Microfluidic mazes provide a useful application to study spatial structure and community dynamics. Mazes can designed to any topology, and they have been used to study bacterial behaviors like chemotaxis, motility, and quorum sensing (56, 66, 67). (B) SCFM2 synthetic cystic fibrosis medium (SCFM2) is a defined liquid growth media that closely mimics the composition of sputum found in the CF lung. *Pa* recovered from patient sputum samples forms free floating aggregates of 10 to 1,000 cells, in comparison to *Pa* cultured in flow cells and other lab media, which often form surface-attached biofilms with a characteristic “mushroom” shape. This system can be utilized to model inter- and intraspecies interactions that may occur at population relevant sizes *in vivo* (44, 52). (C) Micro-3D-printing involves the construction of microscopic containers around single bacterial cells using photolithography. Bacteria are contained to divide and form aggregates of a designed size, allowing diffusion of nutrients and other biologically active molecules such as quorum-sensing signals and antibiotics. The ability to print in any three-dimensional (3D) conformation allows for the creation of tailor-made bacterial ecosystems. (D) Alginate beads are a flexible model for studying microbe behavior and community spatial organization across chemical gradients. *Pa* inoculated into alginate beads forms aggregates that are similar in size to those seen in CF sputum and chronic wounds. In this example, *Pa* aggregates are spatially organized across oxygen and nitrate gradients, where altered respiration rates impact antibiotic tolerance (57). This figure was created with BioRender.com.

There are many models being used to study polymicrobial communities and interactions, and each has its own utilities. While a comprehensive list of models is outside the scope of this minireview, we describe several models that replicate some aspects of *in vivo* conditions in Fig. 2.

The predominant limitation of most models is their ability to mimic the dynamic environments found in human infection sites. A breadth of work from recent decades has shown that bacterial behavior can differ dramatically between planktonic and biofilm modes of growth and between *in vivo* and *in vitro* environments. This difference in behaviors can be shaped by factors in the microenvironment, including chemical gradients, available nutrients, anoxic versus hypoxic conditions, the host immune response, and microbe-microbe community interactions. Closed system models such as agar and broth do have utility for elucidating basic mechanisms and establishing a baseline knowledge. Information gathered from these systems should be reevaluated in a dynamic model that (i) mimics conditions in the human infection site and (ii) facilitates the observation of spatial organization and community dynamics at a high resolution. As new pathogens emerge and bacterial resistance to current medications become commonplace, insights gained from such models will likely drive the development of future therapies.

## FUTURE DIRECTIONS

We believe that future developments in microbiology and antimicrobial therapies will be driven by a fundamental shift in the way we as researchers conceptualize microbial infections. When we examine them within the context of their complex ecosystems and not as just isolated populations of cells, new interactions and behaviors are revealed that have impacts on disease progression and treatment efficacy. Discovering these mechanisms and understanding their implications will be critical. To this end, there are still many questions that remain to be answered. Major topics that we hope to see addressed in the future include understanding the mechanisms by which microbial communities form and maintain themselves, mapping the various ways one community may interact with another, defining how communities interact with the host, and understanding how each can influence disease.

The environment of the cystic fibrosis lung is an ideal model for studying the intricacies of microbial ecosystems because the occurrence rates and succession of common pathogens are fairly defined. However, the emergence of pathogens like *Af* and NTM shows that what we thought we knew about CF is changing and highlights the dynamic nature of microbial communities. An immediate challenge will be developing models that allow us to characterize these communities as they would be found *in vivo.* We need robust models that mimic *in vivo* growth and support observation of spatial orientation and the study of microbes in the context of their habitat.

## References

[1] Cystic Fibrosis Foundation. 2019 Cystic fibrosis 2018 patient registry annual report. Cystic Fibrosis Foundation, Bethesda, MD.

[2] Ramsay KA, Sandhu H, Geake JB, Ballard E, O’Rourke P, Wainwright CE, Reid DW, Kidd TJ, Bell SC 2017 The changing prevalence of pulmonary infection in adults with cystic fibrosis: a longitudinal analysis. J Cyst Fibros 16:70–77. doi:10.1016/j.jcf.2016.07.010. 27515017

[3] Breuer O, Schultz A, Turkovic L, De Klerk N, Keil AD, Brennan S, Harrison J, Robertson C, Robinson PJ, Sly PD, Ranganathan S, Stick SM, Caudri D 2019 Changing prevalence of lower airway infections in young children with cystic fibrosis. Am J Respir Crit Care Med 200:590–599. doi:10.1164/rccm.201810-1919OC. 30811949

[4] Salsgiver EL, Fink AK, Knapp EA, LiPuma JJ, Olivier KN, Marshall BC, Saiman L 2016 Changing epidemiology of the respiratory bacteriology of patients with cystic fibrosis. Chest 149:390–400. doi:10.1378/chest.15-0676. 26203598 PMC5831653

[5] Stacy A, McNally L, Darch SE, Brown SP, Whiteley M 2016 The biogeography of polymicrobial infection. Nat Rev Microbiol 14:93–105. doi:10.1038/nrmicro.2015.8. 26714431 PMC5116812

[6] Rodríguez-Sevilla G, García-Coca M, Romera-García D, Aguilera-Correa JJ, Mahíllo-Fernández I, Esteban J, Pérez-Jorge C 2018 Non-tuberculous mycobacteria multispecies biofilms in cystic fibrosis: development of an *in vitro Mycobacterium abscessus* and *Pseudomonas aeruginosa* dual species biofilm model. Int J Med Microbiol 308:413–423. doi:10.1016/j.ijmm.2018.03.003. 29555180

[7] Scott J, Sueiro-Olivares M, Ahmed W, Heddergott C, Zhao C, Thomas R, Bromley M, Latgé J-P, Krappmann S, Fowler S, Bignell E, Amich J 2019 *Pseudomonas aeruginosa*-derived volatile sulfur compounds promote distal *Aspergillus fumigatus* growth and a synergistic pathogen-pathogen interaction that increases pathogenicity in co-infection. Front Microbiol 10:2311. doi:10.3389/fmicb.2019.02311. 31649650 PMC6794476

[8] Rose SJ, Bermudez LE 2014 *Mycobacterium avium* biofilm attenuates mononuclear phagocyte function by triggering hyperstimulation and apoptosis during early infection. Infect Immun 82:405–412. doi:10.1128/IAI.00820-13. 24191301 PMC3911830

[9] Qvist T, Eickhardt S, Kragh KN, Andersen CB, Iversen M, Høiby N, Bjarnsholt T 2015 Chronic pulmonary disease with *Mycobacterium abscessus* complex is a biofilm infection. Eur Respir J 46:1823–1826. doi:10.1183/13993003.01102-2015. 26493807

[10] Esther CR, Esserman DA, Gilligan P, Kerr A, Noone PG 2010 Chronic *Mycobacterium abscessus* infection and lung function decline in cystic fibrosis. J Cyst Fibros 9:117–123. doi:10.1016/j.jcf.2009.12.001. 20071249 PMC3837580

[11] American Lung Association. 2020 Learn about nontuberculous mycobacteria (NTM). American Lung Association, Chicago, IL.

[12] Chmiel JF, Aksamit TR, Chotirmall SH, Dasenbrook EC, Elborn JS, LiPuma JJ, Ranganathan SC, Waters VJ, Ratjen FA 2014 Antibiotic management of lung infections in cystic fibrosis. II. Nontuberculous mycobacteria, anaerobic bacteria, and fungi. Ann Am Thorac Soc 11:1298–1306. doi:10.1513/AnnalsATS.201405-203AS. 25167882 PMC5469357

[13] Jhun BW, Jung WJ, Hwang NY, Park HY, Jeon K, Kang ES, Koh WJ 2017 Risk factors for the development of chronic pulmonary aspergillosis in patients with nontuberculous mycobacterial lung disease. PLoS One 12:e0188716. doi:10.1371/journal.pone.0188716. 29190796 PMC5708732

[14] Roux AL, Catherinot E, Ripoll F, Soismier N, Macheras E, Ravilly S, Bellis G, Vibet MA, Le Roux E, Lemonnier L, Gutierrez C, Vincent V, Fauroux B, Rottman M, Guillemot D, Gaillard JL, Herrmann J-L, OMA Group 2009 Multicenter study of prevalence of nontuberculous mycobacteria in patients with cystic fibrosis in France. J Clin Microbiol 47:4124–4128. doi:10.1128/JCM.01257-09. 19846643 PMC2786646

[15] Rose SJ, Babrak LM, Bermudez LM 2015 *Mycobacterium avium* possesses extracellular DNA that contributes to biofilm formation, structural integrity, and tolerance to antibiotics. PLoS One 10:e0128772. doi:10.1371/journal.pone.0128772. 26010725 PMC4444313

[16] Stephenson D, Perry A, Appleby MR, Lee D, Davison J, Johnston A, Jones AL, Nelson A, Bourke SJ, Thomas MF, De Soyza A, Lordan JL, Lumb J, Robb AE, Samuel JR, Walton KE, Perry JD 2019 An evaluation of methods for the isolation of nontuberculous mycobacteria from patients with cystic fibrosis, bronchiectasis and patients assessed for lung transplantation. BMC Pulm Med 19:19. doi:10.1186/s12890-019-0781-2. 30665395 PMC6341538

[17] Skolnik K, Kirkpatrick G, Quon BS 2016 Nontuberculous mycobacteria in cystic fibrosis. Curr Treat Options Infect Dis 8:259–274. doi:10.1007/s40506-016-0092-6. 28035194 PMC5155018

[18] Pierre-Audigier C, Ferroni A, Sermet-Gaudelus I, LeBourgeois M, Offredo C, Vu-Thien H, Fauroux B, Mariani P, Munck A, Bingen E, Guillemot D, Quesne G, Vincent V, Berche P, Gaillard J-L 2005 Age-related prevalence and distribution of nontuberculous mycobacterial species among patients with cystic fibrosis. J Clin Microbiol 43:3467–3470. doi:10.1128/JCM.43.7.3467-3470.2005. 16000480 PMC1169165

[19] Floto RA, Olivier KN, Saiman L, Daley CL, Herrmann JL, Nick JA, Noone PG, Bilton D, Corris P, Gibson RL, Hempstead SE, Koetz K, Sabadosa KA, Sermet-Gaudelus I, Smyth AR, van Ingen J, Wallace RJ, Winthrop KL, Marshall BC, Haworth CS 2016 US Cystic Fibrosis Foundation and European Cystic Fibrosis Society consensus recommendations for the management of non-tuberculous mycobacteria in individuals with cystic fibrosis. Thorax 71(Suppl 1):i1–i22. doi:10.1136/thoraxjnl-2015-207360. 26666259 PMC4717371

[20] Kamata H, Asakura T, Suzuki S, Namkoong H, Yagi K, Funatsu Y, Okamori S, Uno S, Uwamino Y, Fujiwara H, Nishimura T, Ishii M, Betsuyaku T, Hasegawa N 2017 Impact of chronic *Pseudomonas aeruginosa* infection on health-related quality of life in *Mycobacterium avium* complex lung disease. BMC Pulm Med 17:198. doi:10.1186/s12890-017-0544-x. 29237500 PMC5727955

[21] Batt SM, Minnikin DE, Besra GS 2020 The thick waxy coat of mycobacteria, a protective layer against antibiotics and the host’s immune system. Biochem J 477:1983–2006. doi:10.1042/BCJ20200194.2005. 32470138 PMC7261415

[22] Taylor JL, Palmer SM 2006 *Mycobacterium abscessus* chest wall and pulmonary infection in a cystic fibrosis lung transplant recipient. J Heart Lung Transplant 25:985–988. doi:10.1016/j.healun.2006.04.003. 16890122

[23] Chandrashekaran S, Escalante P, Kennedy CC 2017 *Mycobacterium abscessus* disease in lung transplant recipients: diagnosis and management. J Clin Tuberc Other Mycobact Dis 9:10–18. doi:10.1016/j.jctube.2017.08.002. 29276785 PMC5737965

[24] Knoll BM, Kappagoda S, Gill RR, Goldberg HJ, Boyle K, Baden LR, Fuhlbrigge AL, Marty FM 2012 Non-tuberculous mycobacterial infection among lung transplant recipients: a 15-year cohort study. Transpl Infect Dis 14:452–460. doi:10.1111/j.1399-3062.2012.00753.x. 22676720

[25] Hoppe JE, Sagel SD 2019 Shifting landscape of airway infection in early cystic fibrosis. Am J Respir Crit Care Med 200:528–529. doi:10.1164/rccm.201903-0529ED. 30875233 PMC6727166

[26] Patterson K, Strek ME 2010 Allergic bronchopulmonary aspergillosis. Proc Am Thorac Soc 7:237–244. doi:10.1513/pats.200908-086AL. 20463254

[27] Verweij PE, Ananda-Rajah M, Andes D, Arendrup MC, Brüggemann RJ, Chowdhary A, Cornely OA, Denning DW, Groll AH, Izumikawa K, Kullberg BJ, Lagrou K, Maertens J, Meis JF, Newton P, Page I, Seyedmousavi S, Sheppard DC, Viscoli C, Warris A, Donnelly JP 2015 International expert opinion on the management of infection caused by azole-resistant *Aspergillus fumigatus*. Drug Resist Updat 21-22:30–40. doi:10.1016/j.drup.2015.08.001. 26282594

[28] Centers for Disease Control and Prevention. 2019. Treatment | Aspergillosis | Types of Fungal Diseases | Fungal Diseases. Centers for Disease Control and Prevention (CDC), Atlanta, GA.

[29] Amin R, Dupuis A, Aaron SD, Ratjen F 2010 The effect of chronic infection with *Aspergillus fumigatus* on lung function and hospitalization in patients with cystic fibrosis. Chest 137:171–176. doi:10.1378/chest.09-1103. 19567494

[30] de Vrankrijker AMM, van der Ent CK, van Berkhout FT, Stellato RK, Willems RJL, Bonten MJM, Wolfs TFW 2011 *Aspergillus fumigatus* colonization in cystic fibrosis: implications for lung function? Clin Microbiol Infect 17:1381–1386. doi:10.1111/j.1469-0691.2010.03429.x. 21087348

[31] Hong G, Alby K, Ng SCW, Fleck V, Kubrak C, Rubenstein RC, Dorgan DJ, Kawut SM, Hadjiliadis D 2020 The presence of *Aspergillus fumigatus* is associated with worse respiratory quality of life in cystic fibrosis. J Cyst Fibros 19:125–130. doi:10.1016/j.jcf.2019.08.008. 31446018 PMC7839066

[32] Cystic Fibrosis Foundation. 2020 Aspergillus and allergic bronchopulmonary aspergillosis. Cystic Fibrosis Foundation, Bethesda, MD.

[33] Hamprecht A, Morio F, Bader O, Le Pape P, Steinmann J, Dannaoui E 2018 Azole resistance in *Aspergillus fumigatus* in patients with cystic fibrosis: a matter of concern? Mycopathologia 183:151–160. doi:10.1007/s11046-017-0162-4. 28653258

[34] Burgel PR, Baixench MT, Amsellem M, Audureau E, Chapron J, Kanaan R, Honoré I, Dupouy-Camet J, Dusser D, Klaassen CH, Meis JF, Hubert D, Paugam A 2012 High prevalence of azole-resistant *Aspergillus fumigatus* in adults with cystic fibrosis exposed to itraconazole. Antimicrob Agents Chemother 56:869–874. doi:10.1128/AAC.05077-11. 22123701 PMC3264284

[35] Lelièvre L, Groh M, Angebault C, Maherault AC, Didier E, Bougnoux ME 2013 Azole resistant *Aspergillus fumigatus*: an emerging problem. Med Mal Infect 43:139–145. doi:10.1016/j.medmal.2013.02.010. 23562488

[36] Reece E, McClean S, Greally P, Renwick J 2019 The prevalence of Aspergillus fumigatus in early cystic fibrosis disease is underestimated by culture-based diagnostic methods. J Microbiol Methods 164:105683. doi:10.1016/j.mimet.2019.105683. 31386863

[37] Minari A, Husni R, Avery RK, Longworth DL, DeCamp M, Bertin M, Schilz R, Smedira N, Haug MT, Mehta A, Gordon SM 2002 The incidence of invasive aspergillosis among solid organ transplant recipients and implications for prophylaxis in lung transplants. Transpl Infect Dis 4:195–200. doi:10.1034/j.1399-3062.2002.t01-2-02002.x. 12535262

[38] Helmi M, Love RB, Welter D, Cornwell RD, Meyer KC 2003 *Aspergillus* infection in lung transplant recipients with cystic fibrosis: risk factors and outcomes comparison to other types of transplant recipients. Chest 123:800–808. doi:10.1378/chest.123.3.800. 12628881

[39] Solé A, Morant P, Salavert M, Pemán J, Morales P, Pastor A, Lozano C, Vicente R, Ramos F, Blasco E, Calvo V, García-Zarza A, Padilla J, Pastor J, Tarazona V 2005 *Aspergillus* infections in lung transplant recipients: risk factors and outcome. Clin Microbiol Infect 11:359–365. doi:10.1111/j.1469-0691.2005.01128.x. 15819861

[40] Viviani L, Harrison MJ, Zolin A, Haworth CS, Floto RA 2016 Epidemiology of nontuberculous mycobacteria (NTM) amongst individuals with cystic fibrosis (CF). J Cyst Fibros 15:619–623. doi:10.1016/j.jcf.2016.03.002. 27050794

[41] Reece E, Segurado R, Jackson A, McClean S, Renwick J, Greally P 2017 Co-colonisation with *Aspergillus fumigatus* and *Pseudomonas aeruginosa* is associated with poorer health in cystic fibrosis patients: an Irish registry analysis. BMC Pulm Med 17:70. doi:10.1186/s12890-017-0416-4. 28431569 PMC5401475

[42] Sass G, Nazik H, Penner J, Shah H, Ansari SR, Clemons KV, Groleau MC, Dietl AM, Visca P, Haas H, Déziel E, Stevens DA 2017 Studies of *Pseudomonas aeruginosa* mutants indicate pyoverdine as the central factor in inhibition of *Aspergillus fumigatus* biofilm. J Bacteriol 200:e00345-17. doi:10.1128/JB.00345-17. 29038255 PMC5717155

[43] Shirazi F, Ferreira JAG, Stevens DA, Clemons KV, Kontoyiannis DP 2016 Biofilm filtrates of *Pseudomonas aeruginosa* strains isolated from cystic fibrosis patients inhibit preformed *Aspergillus fumigatus* biofilms via apoptosis. PLoS One 11:e0150155. doi:10.1371/journal.pone.0150155. 26930399 PMC4773012

[44] Turner KH, Wessel AK, Palmer GC, Murray JL, Whiteley M 2015 Essential genome of *Pseudomonas aeruginosa* in cystic fibrosis sputum. Proc Natl Acad Sci U S A 112:4110–4115. doi:10.1073/pnas.1419677112. 25775563 PMC4386324

[45] Hsieh MH, Lin CY, Wang CY, Fang YF, Lo YL, Lin SM, Lin HC 2018 Impact of concomitant nontuberculous mycobacteria and *Pseudomonas aeruginosa* isolates in non-cystic fibrosis bronchiectasis. Infect Drug Resist 11:1137–1143. doi:10.2147/IDR.S169789. 30127630 PMC6089115

[46] Jorth P, Staudinger BJ, Wu X, Hisert KB, Hayden H, Garudathri J, Harding CL, Radey MC, Rezayat A, Bautista G, Berrington WR, Goddard AF, Zheng C, Angermeyer A, Brittnacher MJ, Kitzman J, Shendure J, Fligner CL, Mittler J, Aitken ML, Manoil C, Bruce JE, Yahr TL, Singh PK 2015 Regional isolation drives bacterial diversification within cystic fibrosis lungs. Cell Host Microbe 18:307–319. doi:10.1016/j.chom.2015.07.006. 26299432 PMC4589543

[47] Harrison F 2007 Microbial ecology of the cystic fibrosis lung. Microbiology (Reading) 153:917–923. doi:10.1099/mic.0.2006/004077-0. 17379702

[48] Ibberson CB, Stacy A, Fleming D, Dees JL, Rumbaugh K, Gilmore MS, Whiteley M 2017 Co-infecting microorganisms dramatically alter pathogen gene essentiality during polymicrobial infection. Nat Microbiol 2:17079. doi:10.1038/nmicrobiol.2017.79. 28555625 PMC5774221

[49] Leung JM, Olivier KN 2013 Nontuberculous mycobacteria: the changing epidemiology and treatment challenges in cystic fibrosis. Curr Opin Pulm Med 19:662–669. doi:10.1097/MCP.0b013e328365ab33. 24048085 PMC6684957

[50] Gilligan PH, Downey DG, Elborn JS, Flume PA, Funk S, Gilpin D, Kidd TJ, McCaughan J, Millar BC, Murphy PG, Rendall JC, Tunney MM, Moore JE 2018 “Pathogen eradication” and “emerging pathogens”: difficult definitions in cystic fibrosis. J Clin Microbiol 56:e00193-18. doi:10.1128/JCM.00193-18. 29875191 PMC6113473

[51] Willner D, Haynes MR, Furlan M, Schmieder R, Lim YW, Rainey PB, Rohwer F, Conrad D 2012 Spatial distribution of microbial communities in the cystic fibrosis lung. ISME J 6:471–474. doi:10.1038/ismej.2011.104. 21796216 PMC3260497

[52] Darch SE, Simoska O, Fitzpatrick M, Barraza JP, Stevenson KJ, Bonnecaze RT, Shear JB, Whiteley M 2018 Spatial determinants of quorum signaling in a *Pseudomonas aeruginosa* infection model. Proc Natl Acad Sci U S A 115:4779–4784. doi:10.1073/pnas.1719317115. 29666244 PMC5939081

[53] Ferreira JAG, Penner JC, Moss RB, Haagensen JAJ, Clemons KV, Spormann AM, Nazik H, Cohen K, Banaei N, Carolino E, Stevens DA 2015 Inhibition of *Aspergillus fumigatus* and its biofilm by *Pseudomonas aeruginosa* is dependent on the source, phenotype and growth conditions of the bacterium. PLoS One 10:e0134692. doi:10.1371/journal.pone.0134692. 26252384 PMC4529298

[54] Caverly LJ, Lipuma JJ 2018 Good cop, bad cop: anaerobes in cystic fibrosis airways. Eur Respir J 52:1801146. doi:10.1183/13993003.01146-2018. 29997183

[55] Einarsson GG, Zhao J, LiPuma JJ, Downey DG, Tunney MM, Elborn JS 2019 Community analysis and co-occurrence patterns in airway microbial communities during health and disease. ERJ Open Res 5:1e0128-17. doi:10.1183/23120541.00128-2017. PMC6612604 31304176

[56] Wessel AK, Hmelo L, Parsek MR, Whiteley M 2013 Going local: technologies for exploring bacterial microenvironments. Nat Rev Microbiol 11:337–348. doi:10.1038/nrmicro3010. 23588251 PMC3984535

[57] Sønderholm M, Kragh KN, Koren K, Jakobsen TH, Darch SE, Alhede M, Jensen PØ, Whiteley M, Kühl M, Bjarnsholt T 2017 *Pseudomonas aeruginosa* aggregate formation in an alginate bead model system exhibits *in vivo*-like characteristics. Appl Environ Microbiol 83:e00113-17. doi:10.1128/AEM.00113-17. 28258141 PMC5394317

[58] Jorth P, Spero MA, Livingston J, Newman DK 2019 Quantitative visualization of gene expression in mucoid and nonmucoid *Pseudomonas aeruginosa* aggregates reveals localized peak expression of alginate in the hypoxic zone. mBio 10:02622-19. doi:10.1128/mBio.02622-19. PMC6918079 31848278

[59] Venkataraman A, Rosenbaum MA, Werner JJ, Winans SC, Angenent LT 2014 Metabolite transfer with the fermentation product 2,3-butanediol enhances virulence by *Pseudomonas aeruginosa*. ISME J 8:1210–1220. doi:10.1038/ismej.2013.232. 24401856 PMC4030227

[60] Phan J, Gallagher T, Oliver A, England WE, Whiteson K 2018 Fermentation products in the cystic fibrosis airways induce aggregation and dormancy-associated expression profiles in a CF clinical isolate of *Pseudomonas aeruginosa*. FEMS Microbiol Lett 365:fny082. doi:10.1093/femsle/fny082. PMC5928460 29617986

[61] Nguyen M, Sharma A, Wu W, Gomi R, Sung B, Hospodsky D, Angenent LT, Worgall S 2016 The fermentation product 2,3-butanediol alters *P. aeruginosa* clearance, cytokine response and the lung microbiome. ISME J 10:2978–2983. doi:10.1038/ismej.2016.76. 27177192 PMC5148197

[62] Bjarnsholt T, Alhede M, Alhede M, Eickhardt-Sørensen SR, Moser C, Kühl M, Jensen PØ, Høiby N 2013 The in vivo biofilm. Trends Microbiol 21:466–474. doi:10.1016/j.tim.2013.06.002. 23827084

[63] Darch SE, Kragh KN, Abbott EA, Bjarnsholt T, Bull JJ, Whiteley M 2017 Phage inhibit pathogen dissemination by targeting bacterial migrants in a chronic infection model. mBio 8:e00240-17. doi:10.1128/mBio.00240-17. 28377527 PMC5380840

[64] Margalit A, Kavanagh K, Carolan JC 2020 Characterization of the proteomic response of A549 cells following sequential exposure to *Aspergillus fumigatus* and *Pseudomonas aeruginosa*. J Proteome Res 19:279–291. doi:10.1021/acs.jproteome.9b00520. 31693381

[65] Briard B, Heddergott C, Latgé JP 2016 Volatile compounds emitted by *Pseudomonas aeruginosa* stimulate growth of the fungal pathogen *Aspergillus fumigatus*. mBio 7:e00219-16. doi:10.1128/mBio.00219-16. 26980832 PMC4807360

[66] Akiyama T, Williamson KS, Schaefer R, Pratt S, Chang CB, Franklin MJ 2017 Resuscitation of Pseudomonas aeruginosa from dormancy requires hibernation promoting factor (PA4463) for ribosome preservation. Proc Natl Acad Sci U S A 114:3204–3209. doi:10.1073/pnas.1700695114. 28270601 PMC5373351

[67] Park S, Wolanin PM, Yuzbashyan EA, Silberzan P, Stock JB, Austin RH 2003 Motion to form a quorum. Science 301:188. doi:10.1126/science.1079805. 12855801

